# The Usefulness of Peer Review for Selecting Manuscripts for Publication: A Utility Analysis Taking as an Example a High-Impact Journal

**DOI:** 10.1371/journal.pone.0011344

**Published:** 2010-06-28

**Authors:** Lutz Bornmann, Hans-Dieter Daniel

**Affiliations:** 1 Professorship for Social Psychology and Research on Higher Education, ETH Zurich, Zurich, Switzerland; 2 Evaluation Office, University of Zurich, Zurich, Switzerland; George Mason University, United States of America

## Abstract

**Background:**

High predictive validity – that is, a strong association between the outcome of peer review (usually, reviewers' ratings) and the scientific quality of a manuscript submitted to a journal (measured as citations of the later published paper) – does not as a rule suffice to demonstrate the usefulness of peer review for the selection of manuscripts. To assess usefulness, it is important to include in addition the base rate (proportion of submissions that are fundamentally suitable for publication) and the selection rate (the proportion of submissions accepted).

**Methodology/Principal Findings:**

Taking the example of the high-impact journal *Angewandte Chemie International Edition* (AC-IE), we present a general approach for determining the usefulness of peer reviews for the selection of manuscripts for publication. The results of our study show that peer review is useful: 78% of the submissions accepted by AC-IE are correctly accepted for publication when the editor's decision is based on one review, 69% of the submissions are correctly accepted for publication when the editor's decision is based on two reviews, and 65% of the submissions are correctly accepted for publication when the editor's decision is based on three reviews.

**Conclusions/Significance:**

The paper points out through what changes in the selection rate, base rate or validity coefficient a higher success rate (utility) in the AC-IE selection process could be achieved.

## Introduction

Reputable scientific journals only publish manuscripts that have been subjected to peer review – that is, critical scrutiny by scientific experts. When a manuscript is submitted, reviewers who are researching and publishing work in the same field (peers) are asked to evaluate the content of the manuscript (e.g., significance and originality of the research findings) and recommend to the editor that the manuscript be published, revised and then published, or rejected [1]. On the basis of these recommendations, the journal editor makes the decision to accept or reject for publication. This means that although experts in a research area are consulted for the reviewing of the manuscript, the reviewing does not include the selection decision on the submissions: Peer review forms the basis for the selection decision made by an editor. The conscientious editor must decide on the validity of peer reviews. “Especially in controversial and highly competitive fields, the responsible editor is called upon to do more than simply average the reviewers' opinions. Reviews may be unavoidably and understandably biased by personal involvement and convergence may occur only slightly more often than expected by chance. The editor must glean what seems objective and logical from the review, and the editor's ability to do so depends on the quality of the review, and on his or her familiarity with the subject of the paper, as well as on a sense of where the paper stands in light of the standards and constituency of the intended audience” [2, p. 40]. The editorial decision can later prove to be successful (if manuscripts are accepted that after publication are useful for the further research in a field) or not (if useful manuscripts are not accepted for publication). The objective of this study is to investigate whether peer review contributes to valid editorial decisions or not.

We have examined in three publications [3], [4], [5] the predictive validity of the selection decisions at the journal *Angewandte Chemie International Edition* (AC-IE). AC-IE is a chemistry journal with a higher annual Journal Impact Factor (JIF) (provided by Thomson Reuters, Philadelphia, PA) than the JIFs of comparable journals (10.879 in the 2008 Journal Citation Reports, Science Edition). It is a journal of the German Chemical Society (Gesellschaft Deutscher Chemiker (GDCh), Frankfurt am Main, Germany) and is published by Wiley-VCH (Weinheim, Germany). The journal introduced peer review in 1982, primarily in conjunction with one of the document types published in the journal, “Communications,” which are short reports on works in progress or recently concluded experimental or theoretical investigations. What the editors of AC-IE look for most of all is excellence in chemical research. Submissions that reviewers deem to be of high quality are selected for publication.

For the investigation of the predictive validity of the AC-IE publication decisions citation counts for the accepted and rejected (but published elsewhere) manuscripts were used. In the absence of other operationalizable criteria, a conventional approach is to use citation counts as a proxy for research quality, since they measure the international impact of scientific work [6]. These analyses showed that on average (arithmetic mean and median), accepted manuscripts have clearly higher citation counts than rejected manuscripts that are published elsewhere [3], [4], [5]. A comparison of average citation counts of accepted and rejected (but published elsewhere) manuscripts with international scientific reference standards found that mean citation counts below baseline values were significantly less frequent for accepted manuscripts than for rejected (but published elsewhere) manuscripts [3]. Both results suggest for AC-IE that the editorial decisions are on average related to the manuscripts' future scientific impact and thus have high predictive validity.

Using an approach developed by Bornmann et al. [7], [ 8], [ see also 9] and, independently of our work, discussed by Straub [10], [11] and Thorngate, Dawes, and Foddy [12], we determined for AC-IE manuscript selection decisions the extent of ‘erroneous’ and ‘correct’ decisions. For erroneous decisions we distinguished type I and type II errors: In type I errors, the editors concluded that a manuscript had the scientific potential for publication and accepted it, when it in fact did not, as reflected in a manuscript's low scientific impact subsequent to publication. In type II errors, the editor concluded that a manuscript did *not* have the scientific potential for publication and rejected it, when it actually did – as reflected in a high scientific impact subsequent to publication. We found that the AC-IE editors' decisions regarding 15% of the manuscripts demonstrate a type I error (accepted manuscripts that did not perform as well as or worse than the average rejected manuscript) [5]. Moreover, the decisions regarding 15% of the manuscripts showed a type II error (rejected manuscripts that performed equal to or above the average accepted manuscript). The large part of the AC-IE editorial decisions (70%) could be classified as either correctly accepted (later highly cited) or correctly rejected (later low citation counts).

In the present study, we expand the approach that we used in previous investigations of the predictive validity of the manuscript selection process at AC-IE to include the model developed by Taylor and Russell [13], in that we included in the analysis the base rate (proportion of manuscripts fundamentally suitable for publication), the selection rate (percentage of manuscripts accepted), and the ratings of the reviewers (upon which the editorial decisions were made). Using this model we are able to determine not only the validity of the editorial decisions (percentage of erroneous and correct decisions) but also (and especially) the usefulness of the peer review for the selection decisions. For example, if a high proportion of manuscripts submitted to a journal show very high quality (measured as the citation counts of these papers after publication) and if a large percentage of these manuscripts are published, then peer review is of only limited utility for the selection decision according to the model developed by Taylor and Russell [13].

### The Taylor and Russell model

The importance of the base rate and selection rate in assessment of a selection process was established by Taylor and Russell [13] in a paper titled, “The relationship of validity coefficients to the practical effectiveness of tests in selection: Discussion and tables.” Taylor and Russell's model has been called “the most well-known utility model” [14, p. 192]. In the paper Taylor and Russell presented a method for determining the success of a personnel selection process that is still used today; in the present study we apply their approach to the success of the selection of submissions to a scientific journal.

The model consists of four parameters [13]. Applied to the manuscript selection process they are:

1. The validity coefficient *r_xy_* indicates the strength of the relationship between the predictor value (x, outcome of peer review) and the criterion value (y, the scientific quality of a submission).
2. The base rate is the percentage of submissions that are ‘qualified‘ submissions. Qualified submissions are papers that are fundamentally suitable for publication. After publication they are comparatively frequently cited.
3. The selection rate is the percentage of submissions that are accepted for publication.
4. The success rate is the percentage of submissions accepted that are qualified submissions. This percentage can either be computed from the available data, as shown in the following, or taken from the tables provided by Taylor and Russell [13], based on certain assumptions.

**Figure 1:**
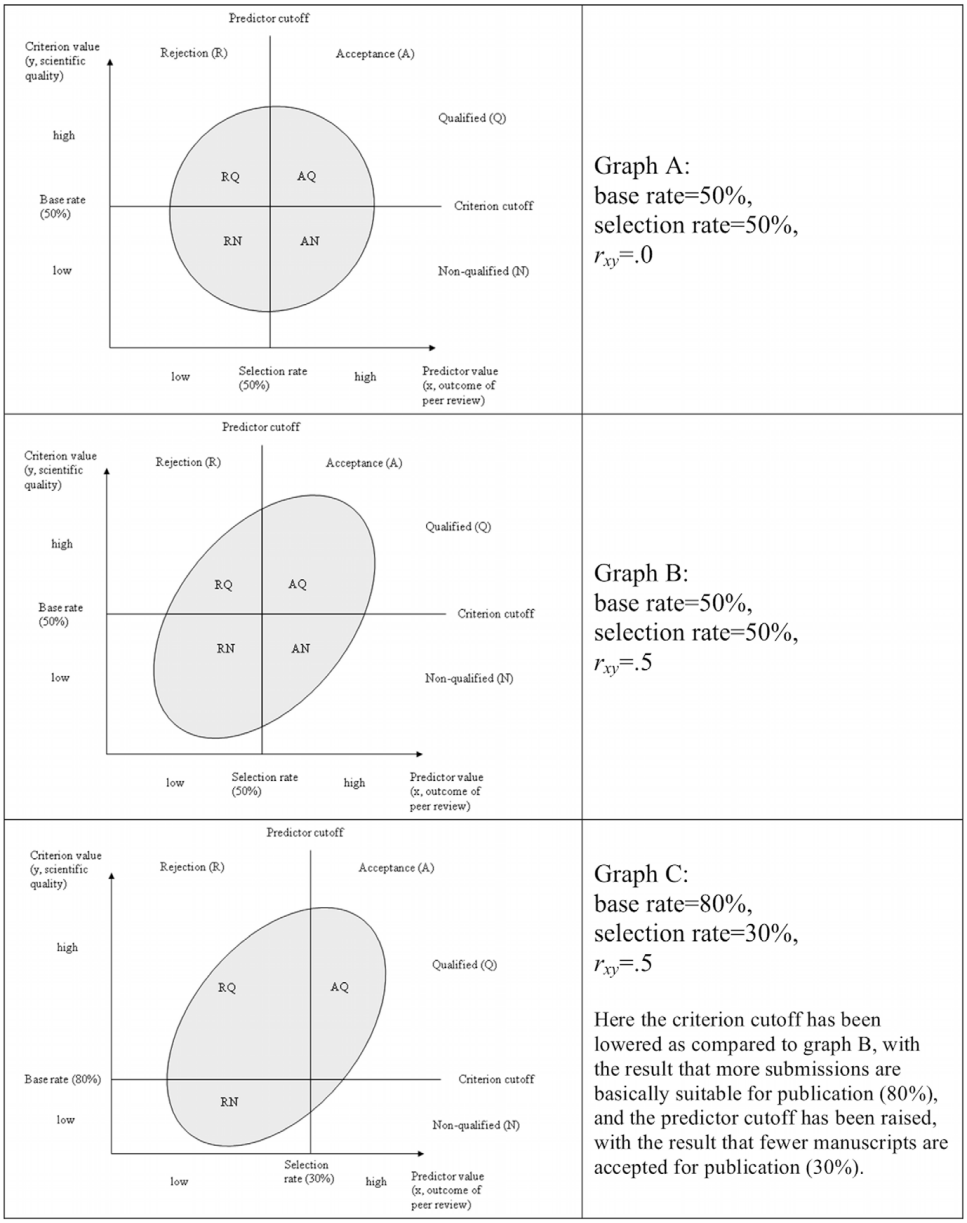
Dependency of the number of accepted and qualified submissions (AQ) on base rate, selection rate and validity coefficient (*r_xy_*).

The three graphs (A, B and C) in Figure 1 show the relationship between the four parameters for a fictitious manuscript selection process. The gray point cloud in the center of each graph represents the validity of the process. On the x-axis is the outcome of peer review (reviewers' ratings) and on the y-axis the scientific quality of a submission (measured as citation counts determined ex post). For every submission this yields a point in the point cloud. If both the predictor and the criterion are normally distributed, a linear relationship between them can be assumed, and if there is a minimum of validity (*r_xy_*>0), the points of all submissions produce a point cloud in the form of an ellipse. However, if *r_xy_* = 1 or *r_xy_* = −1 the result is a line (maximal validity), and if *r_xy_* = 0, the result is a circle (no validity).

Areas AQ and RN in the three graphs of Figure 1 represent correct decisions – that is, if the editors used favorable peer review ratings to select submissions (above the predictor cutoff), those submissions in area AQ would be selected, and after publication they would make a substantial contribution to scientific advancement in a research field (they would be cited with above-average frequency). Those submissions in area RN would be rejected correctly, because they received unfavorable ratings by the reviewers (below the predictor cutoff) and the scientific impact of the manuscripts is low (after publication they would not contribute towards scientific advancement). Areas AN and RQ represent erroneous decisions – those in area AN would be selected because of favorable ratings by the reviewers, but as low quality research they would not contribute towards scientific advancement in a research field (this type of error was called type I error above). Submissions in area RQ would be rejected because of unfavorable ratings by the reviewers, even though they would later turn out to be successful, high impact research (called type II error above).

Based on the number of submissions found in each of the four areas, it is possible to calculate the base rate, selection rate, and success rate:
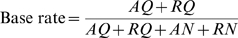
(1)

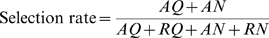
(2)

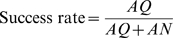
(3)


If there is no correlation between the selection process and the success rate (then the point cloud is a circle) and the base and selection rates are 50% (meaning that one-half of the submissions is qualified and one-half of the submissions is selected), only one-half of the accepted submissions will be qualified (see graph A in Figure 1). This is equivalent to a random selection: The peer reviewing cannot contribute towards increasing the probability that qualified submissions will be accepted. If the point cloud is not a circle but an ellipse, based on peer review more qualified than non-qualified submissions will usually be accepted – if the base and selection rates remain unchanged (see graph B in Figure 1). The higher the validity coefficient (the coefficient for the correlation between the predictor value and the criterion value), the narrower the ellipse is and the more qualified submissions that are accepted. An increase in the validity of a manuscript selection process hence increases the probability that qualified submissions (later highly-cited publications) are selected and unqualified submissions (later publications with low citation counts) rejected, other things being equal [see here 15].

However, the success of a manuscript selection process can also be increased by raising the base rate and thus the number of submissions fundamentally suitable (qualified) for publication. This can be achieved, for example, through successful submission policy (in this case, the point cloud in the graphs would shift upwards). Even if a manuscript selection process is not valid (*r_xy_* = 0, see graph A), as a result there would be more qualified than non-qualified submissions among the accepted submissions. Another possible way to increase the success of a manuscript selection process is to reduce the selection rate. However, this measure is only successful if there is a correlation between the outcome of peer review and the scientific quality of a submission (*r_xy_*>0; that is, in graph A this measure would not result in an improved success rate). If the vertical line (the predictor cutoff) in graph B in Figure 1 is shifted to the right, fewer non-qualified submissions are selected.

Taylor and Russell [13] provided eleven tables from which, based on the given base rate, selection rate, and validity coefficient, the percentage of correctly accepted submissions (AQ/(AQ+AN)) can be taken. For one, the Taylor-Russell tables can be used as an alternative to calculation of the success rate using equation (3). For another, using the tables we can examine how the success rate of a selection process (utility) would be changed by different (hypothetical) values for base rate, selection rate, and/or validity coefficient. Taylor and Russell developed their tables “making use of Pearson's Tables for Finding the Volumes of the Normal Bivariate Surface (1931)” [14, p. 196]. That means the Taylor-Russell tables are appropriate only if “the assumptions of bivariate normal, linear, homoscedastic relationships between predictor and criterion” [14, p. 197] are fulfilled. For instance, with base and selection rates of 50% and a validity coefficient of *r_xy_* = .5 (see graph B in Figure 1), as the Taylor-Russell table shows, 67% of the submissions accepted for publication are correctly accepted (that is, they are qualified submissions).

In contrast to graph B, in graph C in Figure 1 the base rate has been raised to 80% (that means that more submissions are fundamentally qualified for publication) and the selection rate has been reduced to 30% (that means that fewer submissions are selected by the editors). Even though the validity coefficient in graph C is exactly the same as in graph B (*r_xy_* = .5), in graph C submissions will hardly be selected that later turn out to be non-qualified (that is, publications with low impact). According to the corresponding Taylor-Russell table, with a base rate of 80%, a selection rate of 30%, and a validity coefficient of *r_xy_* = .5, 94% of submissions accepted for publication are correctly accepted. However, as graph C shows, also many submissions are rejected that would have been fundamentally suitable for publication.

## Methods

### Manuscript reviewing at AC-IE

A manuscript submitted to AC-IE is usually subject to internal and external review. First, editors at the journal evaluate whether the manuscript contributes to the development of an important area of research (internal review). If the editors find this to be the case, the submitted manuscript is sent to usually three independent reviewers (external review); the reviewers are asked to review it using an evaluation form and a comment sheet. The reviewers know the authors' identities, but their reviews are not signed (single blinding). In the year 2000 the AC-IE evaluation form for reviewers contained the following four questions (in 2008 this was changed to five questions): (1) ‘How important do you consider the results?’ (four response categories: very important; important; less important; unimportant); (2) ‘Do the data obtained by experiment or calculation verify the hypothesis and conclusions?’ (two response categories: yes; no); (3) ‘Is the length of the manuscript appropriate to its contents?’ (three response categories: yes; no - the manuscript is too short; no - the manuscript is too long); (4) ‘Do you recommend acceptance of the Communication?’ (four response categories: yes - without alterations; yes - after minor alterations; yes - but only after major alterations; no). If reviewers find a manuscript unsuitable for AC-IE, they are asked to name another journal in which the study findings might more suitably be published. Once they have received the reviewers' reports, the editors make the decision to accept or reject a manuscript for publication.

Our previous findings show that in general a manuscript is published in AC-IE only if two reviewers rate the results of the study as ‘important’ or ‘very important’ (on question 1 above) and also recommend publication in the journal (do not answer ‘no’ to question 4 above) [16], [17]. The AC-IE calls this their ‘clear-cut rule,’ and the editors use it when they base their publication decisions on either two reviews (one of the reviewers asked to review the manuscript did not do so or did not complete the review on time) or three reviews. If the journal editor makes the decision based on only one review (this was the case for about 100 manuscripts in the present study), a manuscript is accepted for publication only if the reviewer has given positive answers to both of the questions on the evaluation form mentioned above. The editor will make a decision based on only one review if the other reviewers have not sent in their reviews despite several reminders and if the editor is of the opinion that the decision can be made based on one review. For about one-fifth of the manuscripts that were reviewed at AC-IE in the year 2000, the editors (1) requested a review from a so-called top adviser, or (2) had a reviewer review a manuscript that had been revised by the author, or (3) had an appeal reviewed by a reviewer that an author had filed against the rejection of his/her manuscript [17].

Decision rules like the AC-IE's ‘clear-cut rule’ have become a new research topic in recent publications on the journal peer review process [18], [19].

### Database for the present study and conducting of citation analysis

For the investigation of the manuscript selection process at AC-IE, we used information on all 1899 manuscripts that went through internal and external review in the year 2000. Of the 1899 manuscripts, 46% (*n* = 878) were accepted for publication in AC-IE, and 54% (*n* = 1021) were rejected. A search in the literature databases Science Citation Index (SCI) (Thomson Reuters) and Chemical Abstracts (CA) (Chemical Abstracts Services, CAS, Columbus, OH) revealed that of the 1021 rejected manuscripts, 959 (94%) were later published in 136 other (different) journals. For accepted manuscripts and manuscripts that were rejected (but published elsewhere), we determined the number of citations for a fixed time window of three years after the publication year. “Fixed citation windows are a standard method in bibliometric analysis, in order to give equal time spans for citation to articles published in different years, or at different times in the same year” [20, p. 243]]. The citation analyses for the present study were conducted in the year 2007 based on CA. CA is a comprehensive database of publicly disclosed research in chemistry and related sciences (see http://www.cas.org/).

Citation counts are attractive raw data for the evaluation of research output: they are “unobtrusive measures that do not require the cooperation of a respondent and do not themselves contaminate the response (i.e., they are non-reactive)” [21, p. 84]. Although citations have been a controversial measure of both quality and scientific progress [22], they are still accepted as a measure of scientific impact, and thus as a partial aspect of scientific quality [23]: According to van Raan [24], citations provide “a good to even very good quantitative impression of at least one important aspect of quality, namely international impact” (p. 404). For Lindsey, citations are “our most *reliable* convenient measure of quality in science – a measure that will continue to be widely used” [25, p. 201]. In an article by Jefferson, Wager and Davidoff [26] it was pointed out that measuring peer review requires a clear statement of the metric. They identified several, of which several (importance, usefulness and relevancy) mapped to citation counts. In their study, the authors are very explicit about using citation count as their metric.

To find out whether a publication has a high or low citation impact, its performance is compared with international scientific reference standards [27]. For this, Vinkler [28] recommends use of a worldwide reference standard: “Relative Subfield Citedness (R_w_) (where W refers to ‘world’) relates the number of citations obtained by the set of papers evaluated to the number of citations received by a same number of papers published in journals dedicated to the respective discipline, field or subfield” [p. 164, see also 29]. To calculate R_w_ for the manuscripts in this study, specific reference standards were used that refer to the subject areas of CA [30], [31]. CAS categorizes chemical publications into 80 different subject areas (called sections). Every publication becomes associated with a single principal entry that makes clearly apparent the most important aspect of the work [32]. In contrast to the journal sets provided by Thomson Reuters, CA sections are assigned on a paper-by-paper basis [27]. To determine R_w_ in this study, the number of citations for accepted or rejected (but published elsewhere) manuscripts were divided by the (arithmetic) mean number of citations of all publications in a corresponding subject area [see here 33]. According to van Raan [6] the R_w_ quotient allows determination of whether the citation impact of the accepted and rejected (but published elsewhere) manuscripts is far below (R_w_<0.5), below (R_w_ = 0.5–0.8), approximately the same as (R_w_ = 0.8–1.2), above (R_w_ = 1.2–1.5), or far above (R_w_>1.5) the international (primarily the Western world) citation impact baseline for the corresponding subject areas. With R_w_ values above 1.5, the probability of identifying excellent contributions is very high [6].

Of all 1837 manuscripts published in the AC-IE (accepted manuscripts) or another journal (rejected manuscripts), 906 could be included in the analysis of this study. The reduced number was due to missing values for one or more of the variables included in this study. Reviewer's ratings were not available for all manuscripts; for example, some reviewers filled out only the comment sheet and did not fill out the evaluation form with the 2 questions (importance of a manuscript; reviewer's recommendation concerning publication). In addition, for some manuscripts, no citation counts and/or reference values were available [see here 3].

## Results

If the usefulness of peer reviewing for the manuscript selection process is determined using the model developed by Taylor and Russell [13], it must first be established what criterion will be used for considering a submission suitable (qualified) for publication. This can be done only based on the aims of the scholarly journal. As described above, AC-IE has one of the highest JIFs of the journals in the field of chemistry. To guarantee that AC-IE also has a high JIF in future, it is necessary that the editors accept for publication only those submissions that after publication will have far above-average citation counts. For this reason, in this study we rated a submission as qualified for publication in AC-IE, if it showed R_w_>1.5 (criterion cutoff). Hence, all submissions with R_w_>1.5 were categorized as qualified (that is, after publication they made a far above-average contribution to scientific advancement in their subfields) and all submission with R_w_≤1.5 as non-qualified (that is, after publication they do not make this significant contribution to scientific advancement in their subfields).

**Figure 2:**
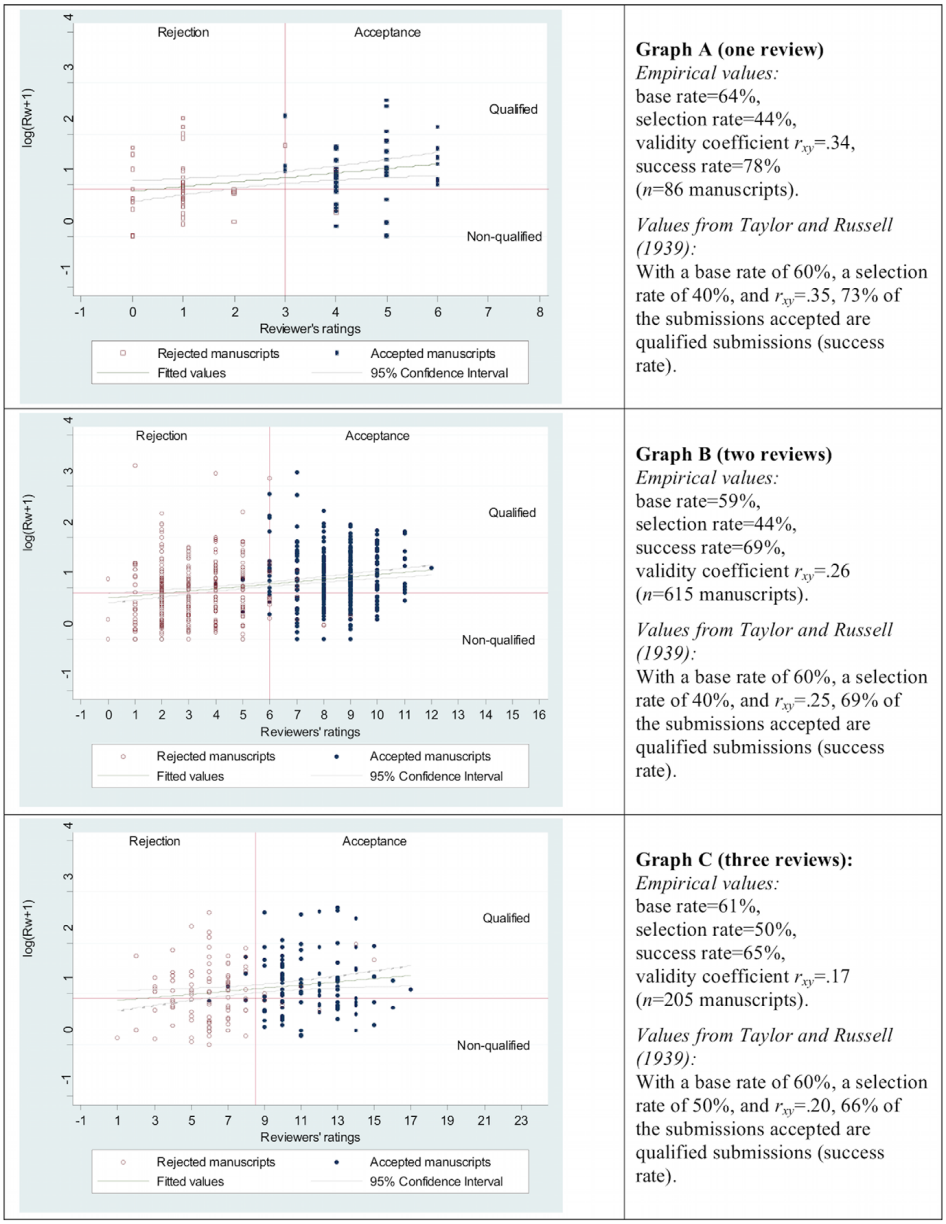
Base rate, selection rate, success rate, and validity coefficient (*r_xy_*) in the reviewing of AC-IE submissions by one (Graph A), two (Graph B), or three (Graph C) reviewers. The green line in the graphs along with 95% confidence interval (gray lines) is the prediction for the criterion, based on a linear regression of the criterion on the predictor.

In the three graphs (A, B, and C) in Figure 2, the R_w_ criterion cutoff is plotted as a red line starting from the y-axis. As the R_w_ values were logarithmized (log(x+1)) in order that the distribution of data more likely approximates a normal distribution [34], the criterion cutoff lies at log(1.5+1) = .92. Graph A shows those submissions for which the AC-IE editor based the publication decision on *one* review; for the submissions in graphs B and C the editor made the publication decisions based on *two* and *three* reviews, respectively. In all three graphs in Figure 2 the ratings of the reviewers that formed the basis for the publication decision are on the x-axis. For these ratings we used the sum of the reviewer's answers to two questions: (1) How important do you consider the results? (four response categories: very important = 3, important = 2, less important = 1, unimportant = 0), and (2) Do you recommend acceptance of the Communication? (four response categories: yes–without alterations = 3; yes–after minor alterations = 2; yes – but only after major alterations = 1; no = 0). For example, if for a submission in graph A a reviewer answered ‘important’ on question (1) and ‘yes – without alterations’ on question (2)‚ the value 5 (2+3) was plotted on the x-axis (reviewer's ratings). If the editor based his/her decision on three reviews, as is the case for the submissions in graph C, we used the sum of the three reviewers' answers on a submission to the two questions: For example, if Reviewer 1 answered ‘less important’ and ‘yes - but after major alterations,’ Reviewer 2 answered ‘unimportant’ and ‘no,’ and Reviewer 3 answered ‘important’ and ‘yes - without alterations,’ then the value 7 (1+1+0+0+2+3) was entered into graph C on the x-axis (reviewers' ratings).

The three graphs show accepted manuscripts as blue circles and rejected manuscripts as red circles. The distributions of the blue and red circles in the graphs make it clearly visible that for nearly all of the manuscripts, values for the reviewers' ratings above a certain predictor cutoff led to acceptance by the AC-IE editor and values below the cutoff led to rejection. When the publication decision is based on one review, the predictor cutoff is 3; when the publication decision is based on two and three reviews, this value is 6 and 8.5, respectively. As the reviewers usually recommend that a submission should be published if it is in their opinion important, the above-mentioned “clear-cut rule” at AC-IE results in most cases in an unambiguous assignment of the reviewers' ratings to the editorial decisions. For only a few manuscripts is this not the case. As presented in Bornmann and Daniel [16], [17], the editor's deviations from the clear-cut rule on few submission can be explained well through further information from the journal's archives (the manuscripts were for example withdrawn by the author during the review process despite a positive review, or the editor suggested to the author that a manuscript be submitted to a specialized journal – the readership of AC-IE are chemists of all subfields).

On the basis of the predictor (reviewers' ratings) and criterion (R_w_) cutoffs, we determined for the submissions in the three graphs of Figure 2 the base and selection rates. Whereas the base rates at AC-IE are 59% (with two reviews), 61% (with three reviews), and 64% (with one review), the selection rates are 44% (with one or with two reviews) and 50% (with three reviews). The resulting validity coefficients (Spearman's rank-order correlations) are *r_xy_* = .34 (with one review), *r_xy_* = .26 (with two reviews), and *r_xy_* = .17 (with three reviews). Given the typical use of two or three reviewers assigned to manuscripts submitted to a journal, we would expect to see that the use of more reviewers per manuscript yields more valid recommendations and thus greater utility. But, the validity coefficients actually drop with more reviews per manuscript. This unexpected result is due to the usual decision process of the AC-IE editors: They wait for further reviews when a publication decision does not seem possible on the received reviews [17]. To test whether more reviews actually lead to a higher coefficient we correlated predictor (reviewers' ratings) and criterion (R_w_) for one review (first review) and two reviews (first and second review) for those 205 manuscripts that have received three reviews. This analyses show the expected order of coefficients: *r_xy_* = .05 (with one review), *r_xy_* = .11 (with two reviews) and *r_xy_* = .17 (with three reviews).

If Eq. (3) is used for calculating the success rates for AC-IE, 78% of the submissions accepted are correctly accepted for publication (with a predictor cutoff>3) when the editor's decision is based on one review, 69% of the submissions are correctly accepted for publication (with a predictor cutoff>6) when the editor's decision is based on two reviews, and 65% of the submissions are correctly accepted for publication when the editor's decision is based on three reviews (with a predictor cutoff>8.5). As the values in Figure 2 show, the percentages of submissions accepted that are qualified submissions are calculated using Eq. (3) differ only a little from the values in the Taylor-Russell tables [13] (even though the assumptions for use of the tables are not completely fulfilled): For the editor's decision based on one review, the Taylor-Russell table percentage is 73% (difference of 5 percentage points), for the editor's decision based on two reviews it is 69% (no difference,) and for editor's decision based on three reviews it is 66% (difference of 1 percentage point).

Independently of the number of reviews on which the editor's decision is based, the percentage of submissions accepted that are qualified submissions is above the base rates; however, the differences between the base rates and success rates are not large. But with base rates of approximately 60% in the AC-IE selection process and validity coefficients of *r_xy_* = .2 and *r_xy_* = .3, respectively, it is difficult, to achieve considerably better success rates than base rates using the predictor (the reviewers' ratings). Using the tables in Taylor and Russell [13], it can then be estimated through what changes in the selection rate, base rate or validity coefficient a higher success rate (utility) in the AC-IE selection process can be achieved. Here the values in the Taylor-Russell tables should be viewed as only rough approximate values, because the assumptions mentioned above for use of the tables are not completely fulfilled. If AC-IE could increase the validity to a value of *r_xy_* = .55 – for example by reviewers having precise information on what makes a manuscript a high quality manuscript (for instance, at AC-IE operationalized as ‘most accessed’ or ‘most cited’ papers, or qualified as ‘Highlights’ by the journal) and information on how high the selection rate at AC-IE is – then according to the Taylor-Russell table, with a base rate of 60% and a selection rate of 40%, there would be 81% submissions accepted that are qualified submissions – an approximately 20 percentage-point gain in utility over the base rate. About the same gain could be achieved through lowering the selection rate to about 5% – with an unchanged validity coefficient and unchanged base rate.

## Discussion

In this paper we presented an approach that in addition to validity points to the importance of the base rate and selection rate for determining the utility of peer review for editors' publication decisions. High predictive validity (meaning a high correlation between the outcome of peer review and the scientific impact of the later publication) does not inevitably mean that the ratings of the reviewers are very useful for the selection of submissions. In addition to considering the base rate and selection rate when evaluating a manuscript selection process, according to Cascio [14] there is further advantage of utility analysis, as it “provides a framework for making decisions by forcing the decision maker to define goals clearly, to enumerate the expected consequences or possible outcomes of his/her decision, and to attach differing utilities or values to each. Such an approach has merit because resulting decisions are likely to rest on a foundation of sound reasoning and conscious forethought” (p. 191).

The Taylor and Russell [13] model basically indicates that an effective submission selection policy should aim to provide for a high base rate, that is, a high percentage of potentially suitable submissions. A good submission policy (such as through providing on a journal's Web site a precise description of what the journal is looking for in a manuscript) can increase the number of suitable manuscripts submitted and decrease the number of unsuitable ones. In other words, the applied policy should lead to self-selection among submissions. Authors should not submit manuscripts that cannot meet the high quality standards of a journal or that deal with a topic that does not fit the journal. This would minimize the required effort/expense for the selection process, to which belong, for example, correspondence with the author (such as acknowledgement of receipt of a submission or rejection notice after review) and peer reviews that turn out to be ‘superfluous.’ This self-selection should be the first step of a manuscript selection process.

The second step should be pre-selection using a pre-screening procedure, so that the later time-consuming and costly process is carried out only for those submissions that seem potentially suitable. A successful pre-selection increases the base rate for the subsequent selection process. For example, the journal *Atmospheric Chemistry and Physics* (ACP) conducts an access review [35], by which the designated reviewers are asked the following questions about whether a manuscript meets the ACP's principal evaluation criteria: (1) scientific significance (‘Does the manuscript represent a substantial contribution to scientific progress within the scope of ACP (substantial new concepts, ideas, methods, or data?’), (2) scientific quality (‘Are the scientific approach and applied methods valid? Are the results discussed in an appropriate and balanced way (consideration of related work, including appropriate references)?’), and (3) presentation quality (‘Are the scientific results and conclusions presented in a clear, concise, and well-structured way (number and quality of figures/tables, appropriate use of English language)?’). The response categories for the three questions are: (1) excellent, (2) good, (3) fair, and (4) poor. Only those manuscripts that are rated at least ‘good’ on all criteria are sent to the referees that already participated in the access review, and perhaps to additional referees, for complete commenting (reviewing). Complete commenting of the manuscript is also not least designed to provide authors suggestions for improvement of the manuscript. Through this procedure, totally unsuitable manuscripts are excluded from the complete commenting stage.

Following similar procedures to those used at ACP, editors and reviewers at other journals could conduct initial pre-screenings of submitted papers to check whether the manuscripts are basically suitable for publication in the journal and whether there are compelling arguments against publication in the journal. The task of the actual review process with extensive commenting would then be to mainly formulate recommendations for improvement of the submitted manuscript and if necessary to recommend a more suitable journal for publication of the manuscript.

The usefulness of peer review for editors' decisions to accept submissions for publication cannot be fully determined using Taylor and Russell's [13] model and tables. For one, use of the Taylor-Russell tables assumes “bivariate normal, linear, homoscedastic relationships between predictor and criterion” [14, p. 197]. If these assumptions are not fulfilled (for bibliometric data it can be generally assumed that these assumptions are not fulfilled completely even after logarithmic transformation), the table values can be used only with reservations. For another, the reviewing of submitted manuscripts in many cases results in improvement of the final publication [32]; this kind of usefulness of peer review is difficult to quantify, however. Regardless of the applicability of the Taylor-Russell tables and the improvement function of peer review, we want to stimulate further studies which test our approach for determining the usefulness of peer reviews for the selection of manuscripts for publication by other journals. It would be especially interesting to base single future studies on more than one journal. This would allow comparisons between the results for different journals.

There are some limitations of our study that we would like to point out: First, we used post-publication above average citation counts as the measure for deeming a submission ‘suitable for publication.’ It can be questioned, whether citation counts is a measure of publication suitability? Recently, Straub [36] offered a number of criteria for high quality research, e.g., logical rigor or replicability of research. Do these criteria relate to, or are reflected in citation counts? Second, we used an ex-post measure (at *t* = 1) to determine the ex-ante suitability of a paper (at *t* = 0). Yet, there are many things that could have happened between *t* = 0 and *t* = 1 that could have contributed to the citation count, without the paper being ‘good.’ For instance, the journal could have risen in attractiveness or ranking, thereby motivating more scholars to cite papers of this journal. Or, the topic of a paper could have been on the peak of a “fashion wave”, i.e., a hot topic [37], even though the paper itself may or may not be a ‘good’ contribution. Third, we chose the thresholds of R_w_> and <1.5 to measure ‘suitability.’ This is an the ‘experience-based’ assignment [6] to impact classes. There might be other ways of measuring ‘suitability’ altogether.

## References

[1] Sense About Science (2004). Peer review and the acceptance of new scientific ideas.

[2] Stossel TP (1985). Refinement in biomedical communication - a case study.. Science Technology & Human Values.

[3] Bornmann L, Daniel H-D (2008). Selecting manuscripts for a high impact journal through peer review: a citation analysis of Communications that were accepted by *Angewandte Chemie International Edition*, or rejected but published elsewhere.. Journal of the American Society for Information Science and Technology.

[4] Bornmann L, Daniel H-D (2008). The effectiveness of the peer review process: inter-referee agreement and predictive validity of manuscript refereeing at *Angewandte Chemie*.. Angewandte Chemie International Edition.

[5] Bornmann L, Daniel H-D (2009). Extent of type I and type II errors in editorial decisions: a case study on *Angewandte Chemie International Edition*.. Journal of Informetrics.

[6] van Raan AFJ, Moed HF, Glänzel W, Schmoch U (2004). Measuring science. Capita selecta of current main issues.. Handbook of quantitative science and technology research The use of publication and patent statistics in studies of S&T systems.

[7] Bornmann L, Daniel H-D (2007). Convergent validation of peer review decisions using the *h* index: extent of and reasons for type I and type II errors.. Journal of Informetrics.

[8] Bornmann L, Wallon G, Ledin A (2008). Does the committee peer review select the best applicants for funding? An investigation of the selection process for two European Molecular Biology Organization programmes.. PLoS One.

[9] Bornmann L, Leydesdorff L, van den Besselaar P (in press). A meta-evaluation of scientific research proposals: different ways of comparing rejected to awarded applications.. Journal of Informetrics.

[10] Straub DW (2008). Type II reviewing errors and the search for exciting papers.. MIS Quarterly.

[11] Straub DW (2008). Thirty years of service to the IS profession: time for renewal at MISQ?. MIS Quarterly.

[12] Thorngate W, Dawes RM, Foddy M (2009). Judging merit.

[13] Taylor HG, Russell JT (1939). The relationship of validity coefficients to the practical effectiveness of tests in selection: discussion and tables.. Journal of Applied Psychology.

[14] Cascio WF (1991). Costing human resources: the financial impact of behavior in organizations.

[15] Cabrera EF, Raju NS (2001). Utility analysis: current trends and future directions.. International Journal of Selection and Assessment.

[16] Bornmann L, Daniel H-D (2009). The luck of the referee draw: the effect of exchanging reviews.. Learned Publishing.

[17] Bornmann L, Daniel H-D (2010). The manuscript reviewing process - empirical research on review requests, review sequences and decision rules in peer review.. Library & Information Science Research.

[18] Schultz DM (in press). Are three heads better than two?.

[19] Egghe L (in press). Study of some editor-in-chief decision schemes..

[20] Craig ID, Plume AM, McVeigh ME, Pringle J, Amin M (2007). Do open access articles have greater citation impact? A critical review of the literature.. Journal of Informetrics.

[21] Smith LC (1981). Citation analysis.. Library Trends.

[22] Bornmann L, Daniel H-D (2008). What do citation counts measure? A review of studies on citing behavior.. Journal of Documentation.

[23] Martin BR, Irvine J (1983). Assessing basic research - some partial indicators of scientific progress in radio astronomy.. Research Policy.

[24] van Raan AFJ (1996). Advanced bibliometric methods as quantitative core of peer review based evaluation and foresight exercises.. Scientometrics.

[25] Lindsey D (1989). Using citation counts as a measure of quality in science. Measuring what's measurable rather than what's valid.. Scientometrics.

[26] Jefferson T, Wager E, Davidoff F (2002). Measuring the quality of editorial peer review.. Journal of the American Medical Association.

[27] Bornmann L, Mutz R, Neuhaus C, Daniel H-D (2008). Use of citation counts for research evaluation: standards of good practice for analyzing bibliometric data and presenting and interpreting results.. Ethics in Science and Environmental Politics.

[28] Vinkler P (1997). Relations of relative scientometric impact indicators. The relative publication strategy index.. Scientometrics.

[29] Vinkler P (1986). Evaluation of some methods for the relative assessment of scientific publications.. Scientometrics.

[30] van Leeuwen TN (2007). Modelling of bibliometric approaches and importance of output verification in research performance assessment.. Research Evaluation.

[31] Neuhaus C, Daniel H-D (2009). A new reference standard for citation analysis in chemistry and related fields based on the sections of Chemical Abstracts.. Scientometrics.

[32] Daniel H-D (1993). Guardians of science. Fairness and reliability of peer review.

[33] Radicchi F, Fortunato S, Castellano C (2008). Universality of citation distributions: toward an objective measure of scientific impact.. Proceedings of the National Academy of Sciences.

[34] Stewart JA (1994). The poisson-lognormal model for bibliometric/scientometric distributions.. Information Processing & Management.

[35] Pöschl U (2004). Interactive journal concept for improved scientific publishing and quality assurance.. Learned Publishing.

[36] Straub DW (2009). Why top journals accept your paper.. MIS Quarterly.

[37] Baskerville RL, Myers MD (2009). Fashion waves in information systems research and practice.. MIS Quarterly.

